# The Association of Peripheral T Lymphocyte Subsets Disseminated Infection by Mycobacterium Tuberculosis in HIV-Negative Patients: A Retrospective Observational Study

**DOI:** 10.3390/medicina58111606

**Published:** 2022-11-07

**Authors:** Qiao Li, Shengsheng Liu, Xiaomeng Li, Ruifang Yang, Chen Liang, Jiajia Yu, Wenhong Lin, Yi Liu, Cong Yao, Yu Pang, Xiaowei Dai, Chuanyou Li, Shenjie Tang

**Affiliations:** 1 Department of Bacteriology and Immunology, Beijing Chest Hospital, Capital Medical University, Beijing 101149, China; 2 Department of Tuberculosis, Anhui Chest Hospital, Hefei 230022, China; 3 Beijing Center for Disease Prevention and Control, Beijing 100035, China; 4 Multidisciplinary Diagnosis and Treatment Centre for Tuberculosis, Beijing Tuberculosis and Thoracic Tumor Research Institute/Beijing Chest Hospital, Capital Medical University, Beijing 101149, China

**Keywords:** tuberculosis, T-lymphocyte subsets, disseminated infection, influence factor

## Abstract

*Background and Objective:* This study was performed to investigate the association of peripheral T lymphocyte subsets with disseminated infection (DI) by *Mycobacterium tuberculosis* (MTB) in HIV-negative patients. *Methods and Materials:* The study included 587 HIV-negative tuberculosis (TB) patients. *Results:* In TB patients with DI, the proportion of CD4+ T cells decreased, the proportion of CD8+ T cells increased, and the ratio of CD4+/CD8+ T cells decreased. According to univariate analysis, smoking, alcohol consumption, rifampicin-resistance, retreatment, and high sputum bacterial load were linked to lower likelihood of developing MTB dissemination. Multivariate analysis indicated that after adjustment for alcohol use, smoking, retreatment, smear, culture, rifampicin-resistance, and CD4+/CD8+, the proportion of CD8+ T cells (but not CD4+ T cells) was independently and positively associated with the prevalence of DI in HIV-negative pulmonary TB (PTB) patients. *Conclusions:* Examining T lymphocyte subsets is of great value for evaluating the immune function of HIV-negative TB patients, and an increase in the CD8+ T cell proportion may be a critical clue regarding the cause of DI in such patients.

## 1. Introduction

Tuberculosis remains a major threat to humanity, with approximately 1.5 million deaths and 9.9 million cases of infection in 2020 [1,2]. It is a disease caused by MTB, which mostly affects the lungs and some extrapulmonary sites, and it is referred to as pulmonary TB (PTB) and extrapulmonary TB (EPTB), respectively [3,4,5,6]. DI, especially miliary PTB complicated by EPTB, is a severe manifestation of TB that significantly contributes to TB-related morbidity and mortality [7,8,9]. For example, tuberculous meningitis is associated with particularly poor outcomes, as it kills or severely disables around 50% of affected patients [10,11,12]. Therefore, further improving the knowledge of miliary TB complicated by EPTB will help identify new strategies for prevention and therapy of TB.

The mechanisms underlying the pathogenesis of TB, specifically disseminated TB, are complex. Cellular immune responses, particularly those involving T cells, have been shown to be crucial for disease onset and progression [13,14]. The first studies correlating the clinical evidence of CD4+ lymphopenia and MTB infection in HIV-negative patients date back to the mid-1980s [15]. Since then, lymphocyte subset modification has been found to be associated with different stages of TB and response to treatment [16,17,18,19]. It suggested that EPTB and disseminated TB are common in immunocompromised patients, and the abundance of Treg cells and activated CD4+ T lymphocytes in patients with prior EPTB was higher than in patients with prior PTB [20,21]. However, the relationship between T lymphocyte subsets and miliary tuberculosis with extrapulmonary dissemination has not been elucidated, especially in countries with a low burden of HIV.

In this study, the distribution of T lymphocyte subsets in HIV-negative TB patients was analyzed, and factors associated with miliary PTB complicated by EPTB were identified using multivariate logistical regression. The results obtained from this study can strengthen our understanding of the changes in immune function in this specific subset of TB patients and can help enable early intervention for high-risk cases in future clinical applications.

## 2. Materials and Methods

### 2.1. Study Population and Ethics Approval

**Figure 1:**
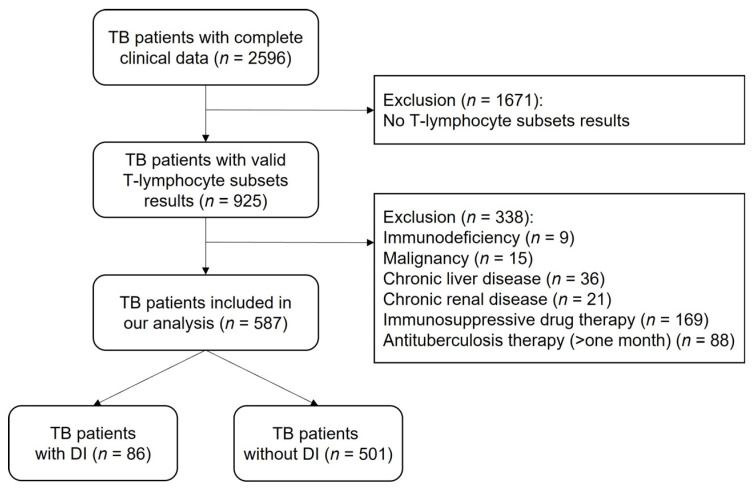
Flowchart of the study population.

This retrospective study analyzed 587 HIV-negative TB patients with complete clinical information who were hospitalized in Beijing Chest Hospital, Beijing, China, and the data were accessed between 1 January 2018 and 31 March 2021. Participants who did not undergo T lymphocyte subset examination or those with immunodeficiency, malignancy, chronic liver disease, chronic renal disease, use of immunosuppressant medications, or currently on antituberculosis therapy for more than one month at the admission date were excluded. The subject screening process is illustrated in Figure 1.

This was an observational retrospective study. Given that all data were collected anonymously, a waiver of consent was approved by the Ethics Committee of Beijing Chest Hospital, Capital Medical University (approval number: YJS-2021-028), which also approved the protocols used in this study.

### 2.2. Case Definitions

PTB was defined by meeting at least one of the following criteria: one positive sputum or bronchoalveolar lavage fluid (BALF) culture; one positive sputum or BALF acid-fast smear and strong radiographic evidence supporting PTB; sputum or BALF with positive GeneXpert MTB/RIF assay results; histopathological changes in the lungs supporting the diagnosis of PTB; typical PTB imaging or immunological findings (such as T-SPOT.TB); and clinical response to anti-TB treatment consistent with PTB.

EPTB was defined by meeting at least one of the following criteria: one culture-positive specimen taken from the lesion site; one culture-positive fluid samples such as pleural effusion, ascetic fluid, or cerebrospinal fluid from the lesion site; a specimen taken from the lesion site with positive GeneXpert MTB/RIF assay results; tissues from the site involved confirmed by pathological analysis; positive response (improvement in clinical manifestations and pulmonary radiology) to anti-TB treatment.

Miliary TB has pulmonary lesions but without extrapulmonary manifestations, and miliary TB complicated by EPTB in this study is referred to as TB with DI. In addition to meeting the diagnosis criteria for PTB and EPTB above, the following criteria were used: imaging revealed a mixture of both sharply and poorly defined (<2 mm) nodules that were widely disseminated throughout the lungs.

### 2.3. Lymphocyte Subset Assays

Venous blood was collected into ethylenediaminetetraacetic acid (EDTA) collection tubes under aseptic conditions, and it was thoroughly mixed with the anticoagulant. Within 24 h after collection, 100 µL of the sample was stained with 20 µL of fluorescent monoclonal antibodies (CD3/CD4/CD8/CD56/CD16/CD19/CD45) (BD Biosciences, San Diego, CA, USA), and the mixture was incubated for 15 min at room temperature in the dark. Next, 450 µL of hemolysin was added to the above-mentioned mixture and mixed again on a vortexer and then incubated for 10 min at room temperature in the dark. Lastly, lymphocyte subsets were assessed within 24 h after staining using the LSRFortessa flow cytometer and analyzed by the BD FACSDiva 8.0.2 software (BD Biosciences, San Diego, CA, USA) according to the manufacturer’s recommendations.

### 2.4. Data Management and Statistical Analysis

From patient medical records, information was obtained pertaining to clinical characteristics of TB inpatients, including demographics, body-mass index (BMI), smoking history, drinking history, diabetic history, Bacillus Calmette-Guérin (BCG) vaccination status, previous TB treatment history, bacteriology, imaging, pathology, albumin, lymphocyte subsets, etc. Data was entered in MS Office Excel (Microsoft, Redmond, WA, USA) datasheets.

The data were expressed as median (interquartile range, IQR) or number (%) according to the nature of the variable. Statistical analysis was performed using GraphPad Prism 8 (GraphPad Software, Inc., La Jolla, CA, USA) or SPSS 24.0 (Chicago, IL, USA). Comparison of T-lymphocyte subsets was conducted using the Mann–Whitney test. Univariate and multivariable logistic analyses were performed to evaluate factors associated with DI in TB patients. Univariate analyses were performed using the chi-square test. Variables that were found to be significant risk factors in univariate analyses were entered simultaneously in the multivariable model (“Enter” method). Odds ratios and 95% confidence intervals for risk were calculated. A *p* < 0.05 was considered statistically significant.

## 3. Results

### 3.1. Participant Characteristics

A total of 587 eligible TB patients were included in the study; 66.4% were male, and the mean age was 48 years old (range: 29–63 years) (Figure 1, Table 1). Other characteristics, including BMI, history of tobacco and alcohol use, diabetic history, BCG vaccination status, history of anti-TB treatment, number of symptoms, bacillary load in the sputum, rifampicin resistant status, and serum albumin level, are also shown in Table 1.

### 3.2. Comparison of T Lymphocyte Subsets between TB Patients with and without DI

**Figure 2:**
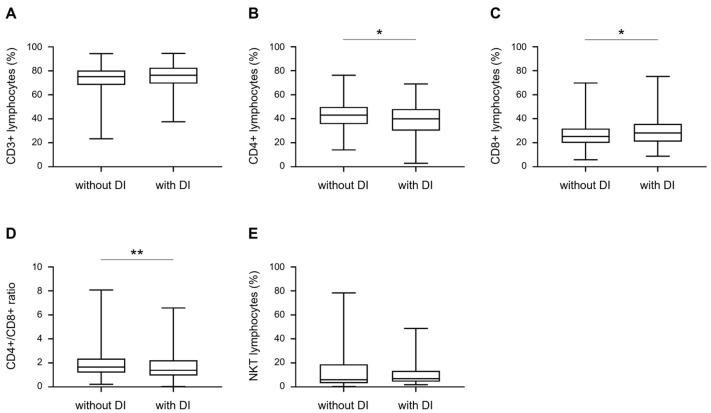
Comparison of T lymphocyte subsets between TB patients with DI and without DI. (**A**–**E**) CD3+, CD4+, CD8+, CD4+/CD8+ ratio, and NKT levels in 86 TB patients with DI and 501 TB patients without DI. Differences between groups were determined using the Mann–Whitney test, * *p* < 0.05, ** *p* < 0.01.

**Figure 3:**
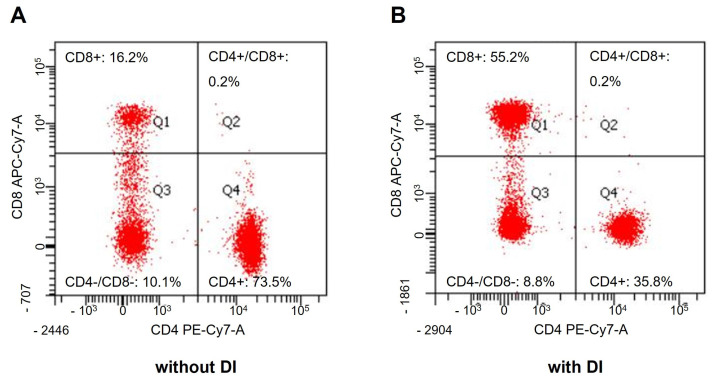
Representative flow cytometry images after CD4+/CD8+ immunostaining. (**A**) The per-centage of CD4+ and CD8+ T cells in TB patients without DI. (**B**) The percentage of CD4+ and CD8+ T cells in TB patients with DI.

We next investigated the levels of peripheral blood lymphocyte subsets in TB patients with and without DI. As shown in Figure 2 and Figure 3, the percentage of CD4+ T cells was significantly lower in TB patients with DI than in those without DI, whereas the percentage of CD8+ T cells was comparably higher. The ratio of CD4+/CD8+ T cells in TB patients with DI was significantly lower than in those without DI. CD3+ and NKT cells were not significantly different between the two groups (Figure 2).

### 3.3. Univariate Analysis of the Other Factors Associated with DI

Various clinical parameters in TB patients were compared between those with and without DI using univariate analysis. Compared with TB patients without DI, TB patients with DI tended to smoke less, consume a lower amount of alcohol, and develop less resistance to rifampicin (Table 2). The proportion of retreatment cases of TB patients with DI was lower than that of patients without DI, and the bacterial load in the sputum was also lower (Table 2).

### 3.4. Multiple Logistic Regression Analysis of the Factors Associated with DI

Factors with univariate analysis *p* value < 0.05 were used in further multivariate analysis, and the results are presented in Table 3. Model 1, which included CD4+ (%), alcohol use, smoking, retreatment, smear, culture, rifampicin resistance, and CD4+/CD8+ as independent variables, showed that retreatment, smear, and rifampicin resistance emerged as significant and independent factors associated with the presence of DI. However, CD4+ (%) was not significantly associated with the presence of DI (*p* = 0.067). Model 2, in which CD8+ (%) was replaced with CD4+ (%), demonstrated that CD8+ (%) was significantly associated with the presence of DI (*p* = 0.026). Furthermore, even when CD4+ (%) and CD8+ (%) were simultaneously included as independent variables in model 3, the addition of CD4+ (%) did not affect the significant association between CD8+ (%) and the presence of DI (*p* = 0.022).

## 4. Discussion

Few prior studies have explored the association of peripheral T lymphocyte subsets with TB with DI in HIV-negative patients. In this study, by comparing and analyzing T lymphocytes, we characterized changes in the proportion of CD4+ and CD8+ T cells as well as in the CD4+/CD8+ ratio in HIV-negative TB patients with DI. Furthermore, we performed logistic regression analysis and found retreatment, smear, rifampicin-resistance, and CD8+ (%) to be independent factors that influence MTB dissemination.

It has been shown that T cells play a critical role in the host immune response against MTB infection [14]. Hosts deficient in CD4+ T cells, particularly those that are immunocompromised, have dramatically increased susceptibility to both primary and reactivation TB [22,23]. This results in higher incidence of disseminated TB, lack of seropositivity, and characteristic imaging findings that may delay diagnosis and superimpose opportunistic infections, all of which may affect treatment outcomes [24]. One of the definite roles of CD4+ T cells in anti-TB immunity is to evolve into Th1 effector cells and produce IFN-γ and TNF-α to directly activate macrophages to control infection or kill MTB-infected macrophages [25]. The Th1 response is a characteristic of protective immunity, while the Th2 response seems to have a counter-regulatory effect. Miliary TB is probably characterized by an imbalance between Th1 and Th2 immune responses, with a predominant shift towards Th2 reactions [26,27]. Our analysis found that the percentage of CD4+ T cells was significantly lower in TB patients with DI than in those without DI, which is consistent with this theory. Another reason for the decrease in CD4+ T cells in patients with DI may be the accumulation of peripheral blood T lymphocytes in the lung parenchyma or in severely infected sites outside the lung [26]. Of course, the specific reasons need to be further studied.

In the immune response to MTB, CD8+ T cells were initially considered to be less important than CD4+ T cells, although it is now recognized that they play an important role [28,29]. For example, CD8+ T cells can also produce cytokines (including IFN-γ, IL-17, IL-2, and TNF) that are assumed to be important for control MTB infection [30]. However, studies on the changes in peripheral blood CD8+ T lymphocytes in TB patients were controversial. Rozot et al., reported that MTB-specific CD8+ T cell responses were detected in the majority of active TB patients (69.8%) and in few latent tuberculosis infection (LTBI) subjects [31]. In contrast, Li et al. showed that CD8+ T cell counts decreased in active TB patients [32]. Our study showed that the proportion of CD8+ T cells was higher in TB with DI than TB without DI. Possible reasons for this discrepancy may include differences in study populations, geographical environments, and ethnic backgrounds. The selection of CD8+ T cell count or its proportion as the study indicator may be another reason for the discrepancy. Although relatively few reports have been made regarding the association between CD8+ T cell levels and MTB dissemination, it has been shown to correlate with disease severity [32]. A possible reason for these findings could be an increase in some regulatory cytokines produced by CD8+ T cells, such as IL-10 and TGF-β, which may result in greater susceptibility to MTB infection [33]. However, the specific mechanisms need to be further defined. The percentage of CD8+ T cells may not change or may be significantly increased, which is due to the marked decrease in the percentage of CD4+ T cells. In the present study, the increase in the proportion of CD8+ T cells in TB with DI indicates that these patients’ immune status is worse and their prognosis is poor, meaning prompt immune intervention is necessary. In addition, it is important to note that the relationship between CD8+ T cells and MTB infection is complex and may be related to different infection sites and stages. Such factors need to be further studied.

Our analyses suggested that sputum smear positive, rifampicin-resistant, and retreatment patients were less prone to MTB dissemination. In patients with disseminated TB, MTB tends to spread through the blood and lymphatic system, and the low bacterial load in the lungs and bronchus leads to a low sputum smear positive rate, which is one of the reasons for the delayed diagnosis of disseminated TB. Rifampicin-resistant strains were found to have stronger intracellular viability, but the invasion and replication efficiencies were lower than those of susceptible strains [34]. Therefore, drug-resistant strains are less likely to cause dissemination than susceptible strains. The proportion of drug resistance in retreatment patients is relatively high, which may partly explain why retreatment patients are not prone to MTB dissemination.

We constructed three models for the logistic regression to further analyze. In addition to including the other independent variables, Models 1 to 3 included CD4+ (%) and CD8 (%) separately and together. The results showed that CD8+ (%), but not CD4+ (%), was independently and significantly associated in a positive manner with DI after adjustment for alcohol use, smoking, retreatment, smear, culture, rifampicin resistance, and CD4+/CD8+. Our results suggest that if the proportion of CD8+ T lymphocytes in peripheral blood of patients with PTB increases, the possibility of miliary TB and MTB dissemination should be considered. Clinicians should consider the possibility of EPTB for early diagnosis and treatment to improve curative effects and prognosis.

Our study had some limitations. First, it was an observational study; only patients admitted to the hospital were enrolled, which might lead to selection bias. Additionally, only venous blood samples were assessed, but comparison with results analyzed from T lymphocytes isolated from whole blood and peripheral blood would make a strong contribution to this study. Second, we did not measure absolute counts of lymphocyte subsets because the clinical significance of detection of percentages and absolute numbers of lymphocyte subsets are different. Finally, follow-up was not performed, and lymphocyte subset recovery in peripheral blood of patients after anti-tuberculous treatment could thus not be evaluated.

## 5. Conclusions

In this study, examination of T lymphocyte subsets enabled greater understanding of the immune function of HIV-negative TB patients. After adjustment for alcohol using, smoking, retreatment, smear, culture, rifampicin-resistance, and CD4+/CD8+, the proportion of CD8+ T cells (but not CD4+ T cells) was independently and positively associated with the prevalence of DI in HIV-negative PTB patients.

## Figures and Tables

**Table 1:** Characteristics of the study participants.

Characteristics	Total Cases (*n* = 587)		Value
	Missing Cases	Valid Cases	
Male gender, No. (%)	0	587	390 (66.4)
Median age (IQR), years	0	587	48 (29–63)
BMI < 18.5, No. (%)	127	460	123 (26.7)
Alcohol use, No. (%)	0	587	155 (26.4)
Cigarette smoking, No. (%)	0	587	232 (39.5)
BCG vaccination, No. (%)	0	587	384 (65.4)
Diabetes, No. (%)	0	587	117 (19.9)
Retreatment *, No. (%)	0	587	186 (31.7)
Number of symptoms ≥4, No. (%)	0	587	243 (41.4)
Smear, positive, No. (%)	0	587	256 (43.6)
Culture, positive, No. (%)	46	541	334 (61.7)
Rifampicin resistant, No. (%)	0	587	164 (27.9)
Albumin decreased, No. (%)	0	587	310 (52.8%)

* Retreated cases refer to anti-tuberculosis treatment ≥1 month in the past. IQR, inter quartile range; BMI, body-mass index; BCG, Bacillus Calmette-Guérin.

**Table 2:** Univariate analysis of DI in TB patients.

Factor	Without DI	With DI	*p* Value
Male gender, No. (%)	339 (67.7)	51 (59.3)	0.129
Age ≥ 60, No. (%)	153 (30.5)	23 (36.7)	0.478
BMI < 18.5, No. (%)	106 (26.2)	17 (30.9)	0.456
Alcohol use, No. (%)	141 (28.1)	14 (16.3)	0.021
Cigarette smoking, No. (%)	211 (42.1)	21 (24.4)	0.002
BCG vaccination, No. (%)	321 (64.1)	63 (73.3)	0.098
Diabetes, No. (%)	104 (20.8)	13 (15.1)	0.226
* Retreatment, No. (%)	180 (35.9)	6 (7.0)	<0.001
Number of symptoms ≥ 4, No. (%)	202 (40.3)	41 (47.7)	0.201
Smear, positive, No. (%)	241 (48.1)	15 (17.4)	<0.001
Culture, positive, No. (%)	306 (64.0)	28 (44.4)	0.003
Rifampicin resistant, No. (%)	160 (31.9)	4 (4.7)	<0.001
Albumin decreased, No. (%)	259 (51.7)	51 (59.3)	0.192

* Retreated cases refer to anti-tuberculosis treatment ≥1 month in the past. BMI, body-mass index; BCG, Bacillus Calmette-Guérin.

**Table 3:** Multiple logistic regression analysis of the factors associated with DI.

Factor	Model 1			Model 2			Model 3		
	OR	95% CI	*p* Value	OR	95% CI	*p* Value	OR	95% CI	*p* Value
Alcohol use									
No	Reference			Reference			Reference		
Yes	0.760	0.320–1.800	0.532	0.753	0.317–1.788	0.520	0.789	0.331–1.879	0.593
Cigarette smoking									
No	Reference			Reference			Reference		
Yes	0.767	0.367–1.601	0.480	0.766	0.365–1.608	0.481	0.739	0.350–1.557	0.426
* Retreatment									
No	Reference			Reference			Reference		
Yes	0.297	0.118–0.749	0.010	0.316	0.126–0.793	0.014	0.305	0.120–0.777	0.013
Smear									
Negative	Reference			Reference			Reference		
Positive	0.373	0.175–0.794	0.010	0.355	0.165–0.761	0.008	0.357	0.166–0.768	0.008
Culture									
Negative	Reference			Reference			Reference		
Positive	0.786	0.405–1.524	0.476	0.841	0.432–1.636	0.610	0.788	0.403–1.541	0.486
Rifampicin resistant									
No	Reference			Reference			Reference		
Yes	0.269	0.091–0.799	0.018	0.237	0.080–0.697	0.009	0.261	0.088–0.774	0.015
CD4+ %	0.968	0.936–1.002	0.067				0.967	0.934–1.001	0.054
CD8+ %				1.041	1.005–1.079	0.026	1.041	1.006–1.077	0.022
CD4+/CD8+ ratio	1.204	0.846–1.713	0.303	1.267	0.894–1.794	0.183	1.602	1.077–2.383	0.020

* Retreated cases refer to anti-tuberculosis treatment ≥1 month in the past. OR, odds ratio; CI, confidence interval.

## Data Availability

Not applicable.
